# Possible Epigenetic Role of Vitexin in Regulating Neuroinflammation in Alzheimer's Disease

**DOI:** 10.1155/2020/9469210

**Published:** 2020-03-09

**Authors:** M. A. F. Yahaya, S. Z. I. Zolkiffly, M. A. M. Moklas, H. Abdul Hamid, J. Stanslas, M. Zainol, M. Z. Mehat

**Affiliations:** ^1^Department of Human Anatomy, Faculty of Medicine & Health Science, Universiti Putra Malaysia, 43400 Serdang, Selangor, Malaysia; ^2^Department of Medicine, Faculty of Medicine & Health Science, Universiti Putra Malaysia, 43400 Serdang, Selangor, Malaysia; ^3^Herbal Medicine Research Centre, Institute for Medical Research, Jalan Pahang, Kuala Lumpur, Malaysia

## Abstract

Alzheimer's disease (AD) has been clinically characterized by a progressive degeneration of neurons which resulted in a gradual and irreversible cognitive impairment. The accumulation of A*β* and *τ* proteins in the brain contribute to the severity of the disease. Recently, vitexin compound has been the talk amongst researchers due to its pharmacological properties as anti-inflammation and anti-AD. However, the epigenetic mechanism of the compound in regulating the neuroinflammation activity is yet to be fully elucidated. Hence, this review discusses the potential of vitexin compound to have the pharmacoepigenetic property in regulating the neuroinflammation activity in relation to AD. It is with hope that the review would unveil the potential of vitexin as the candidate in treating AD.

## 1. Introduction

Alzheimer's disease (AD) was first described by Alois Alzheimer and his coworker named Emil Kraepelin in 1906 at the 37^th^ meeting of the Society of Southwest German Psychiatrists in Tübingen, Germany. They reported to have had a female patient, Auguste D., whom suffered from paranoia, progressive sleep and memory disturbance, aggression, and confusion [1].

Not only the daily life of AD patients but also the people around them will be affected as every AD patient requires close attention in order to maintain the patients' quality of life (QoL) [2]. A comprehensive study conducted by Barbe et al. on the factors that contribute to the QoL of AD patients as well as their caregiver has shown that depression strongly influences the lowest rate of QoL for both AD patients and the caregiver [3]. In the perspective of AD patients, depression is developed due to the need for many medication intake that stems from the presence of multiple comorbidities as well as the patients' perception on their health. On the other hand, in the perspective of AD caregivers, depression is developed as a result of seeing their loved ones suffering from several comorbid disease.

It is estimated that 5.7 million people are suffering from AD in the United States of America (USA) and the number of people that will be affected by the disease is estimated to be 13.8 million people by 2050 [4]. In Malaysia, the prevalence of the disease is estimated to be at 0.454% in 2050 [5] due to the family members that perceived the symptoms of AD as normal aging and hence did not seek for suitable medical treatments [6].

At present, only five drugs have been approved for AD treatment. Such drugs are donepezil, galantamine, rivastigmine, tacrine, and memantine. Donepezil, galantamine, rivastigmine, and tacrine are said to function as cholinesterase inhibitors. Conversely, memantine functions as a glutamate receptor antagonist. However, none of these available drugs show high efficiency in treating AD [7].

AD is a type of complex neurodegenerative disease which is clinically characterized by a progressive degeneration of neurons that resulted in gradual and irreversible cognitive impairment and late dementia [8]. Neuropathologically, the disease has been characterized by gross atrophy of the degeneration of cortical gyrification of the brain and accumulation of both extracellular amyloid plaques and intracellular neurofibrillary tangles [9, 10].

At the early stage of the disease, a patient with AD is normally being diagnosed with a mild cognitive impairment. Histopathologically, neuritic plaques, neurofibrillary tangles, and loss of basal forebrain cholinergic neurons are also being characterized as the early stage of the disease. As the disease progresses, the senile plates and neurofibrillary tangles will be formed as a result of the accumulation of amyloid-*β* (A*β*) and hyperphosphorylation of the tau (*τ*) protein [11]. As a consequence, a patient with AD will suffer from neuronal degeneration and loss of synapses which eventually will lead to severe memory impairment, dementia, and functional decline [12].

A growing number of researches have suggested that the pathogenesis of Alzheimer's disease is not specifically to the neuronal compartment per sé. However, it is also due to the immunological interaction in the brain [13]. Such immunological interaction is triggered from the accumulation of certain proteins that bind to the surface receptors located on the astroglia and microglia cells which eventually causes the release of inflammatory mediators in its environment. Hence, the intention of this review is not only to dissect the epigenetic mechanism in neuroinflammation during the development of Alzheimer's disease but also to discover the possibility of vitexin compound to epigenetically regulate the progression of Alzheimer's disease.

## 2. Neuroinflammation in Alzheimer's Disease

The involvement of inflammation in the pathogenesis of Alzheimer's disease has been initially described more than 20 years ago [14]. Inflammation normally takes place in pathologically vulnerable regions of the brain of an AD patient which is due to the degeneration of tissue and the accumulation of insoluble materials [14]. For the latter, A*β* has been known to be one of the key pathological hallmarks of AD that can induce the inflammation in the brain due to its insoluble property.

**Figure 1:**
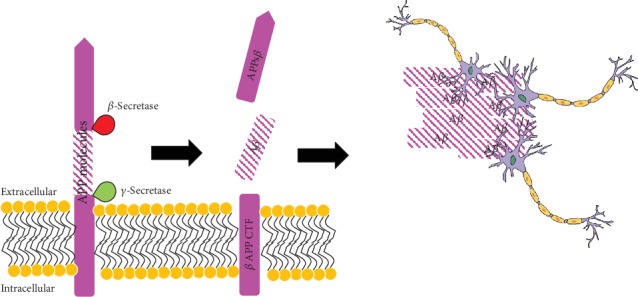
The generation and accumulation of A*β* from APP by proteolytic process via the amyloidogenic pathway which later will inhibit the normal function of neuron cells (adapted and modified from [16]).

A*β* is generated through the sequential proteolytic process of the amyloid precursor protein (APP) via the amyloidogenic pathway [15]. The amyloidogenic pathway (Figure 1) starts with the cleavage of APP molecules by *β*-secretase that resulted in the generation of a large portion of the ectodomain of APP (APPs*β*) and APP carboxy-terminal fragment (*β*APP CTF). The latter will then be cleaved by *γ*-secretase which normally takes place within the hydrophobic environment of biological membranes [16]. The result from the proteolytic process of APP is the synthesis of peptides consisting of 40 to 42 amino acids in length. These overly produced peptides will start to accumulate which subsequently will be deposited within the brain to form diffused and condensed core amyloid plaques which later caused brain inflammation [17]. The accumulation of A*β* normally takes several years and eventually causes *τ* tangles and cognitive decline over a decade or more [18].

The deposition of A*β* in the brain parenchyma will induce the microglial cells that surround it to undergo classical activation [19, 20]. According to Tay et al., microglial cells are the resident “macrophages” and a self-renewing population of myeloid cells which reside in the brain during the development of the embryo [21]. In addition, the cells act as innate immune cells responsible in maintaining the homeostasis of the brain. Despite being known as having the macrophage-like role, microglial cells have now been recognised for their active involvement in influencing synaptic connections in the development of the adult mammalian central nervous system (CNS) [22].

Microglial cells have the tendency to undergo classical activation once they get induced by a stimulus (e.g., lipopolysaccharides (LPS), A*β*) that is present in its environment. The classical activation undergone by microglial cells will cause the cells to phenotypically transform into M1-like macrophages which is analogous to the polarisation of T-helper 1 (Th1) [23, 24]. An extensive review on macrophage and its plasticity feature can be found in an article written by Yahaya et al. [17].

In a normal brain, the microglial cell operates in a protective manner against AD via the classical activation of the triggering receptor expressed in myeloid cells-2 (TREM2) [25]. Once TREM2 is activated, it will initiate the signal transduction pathways that promote chemotaxis, phagocytosis, survival, and proliferation of microglial cells [26, 27]. TREM2 is essential for microglial cells to undergo phagocytosis of apoptotic neurons, bacteria, and A*β* [28, 29].

However, in the condition of neurodegeneration, microglia rather act as an enhancer for the neuroinflammation which eventually leads to more cell deaths [25]. Microglial cells would temporally and spatially activate with the spread of A*β* and *τ* pathology [30]. The number of microglial cells is having a direct proportional relationship with the dimension of the A*β* plaques in AD brain [31]. The death of neurons is caused by the release of excessive inflammatory cytokines such as interleukin- (IL-) 1*β*, IL-6, and tumor necrosis factor-*α* (TNF-*α*) which can as well cause toxicity to the neuron cells and eventually lead to neuroinflammation [32].

In a transgenic AD mouse model study conducted by Stalder et al. [33], they have investigated the relationship between the activated microglial cells with the A*β* plaques in the brains of adult and aged APP23 transgenic mice. From the study, they found that the neuron-derived *β*PP is sufficient to induce both A*β* plaque formation and amyloid-associated microglial activation [34].

Further analysis in the same setting was done by Stalder et al. [34] to investigate the 3D reconstruction of microglial cells in the environment of a dense A*β* plaque. The result from their study showed that the A*β* fibrils are found accumulated extracellularly which leads to the serial of finger-like processes with the widely branched microglial cytoplasm [35]. The result is also parallel with the result obtained by Frautschy et al. where the microglial cells were found to be rapidly proliferated at the periphery site of the A*β* deposits [36].

A*β* is not the only causative agent that induces neuroinflammation, as the overly expressed proinflammatory cytokines secreted by microglial cells also contribute to the same effect. As such, microglial cells have the tendency to become less efficient in executing its normal function (i.e., maintaining the homeostasis of the brain) as we age [33]. The beneficial and detrimental characteristics of microglia in plaque-related neuropathology can be found in the previous genome-wide association studies (GWAS) [17, 37]. In the following section, the epigenetic regulation of neuroinflammation development will be further described and discussed.

## 3. Epigenetic Regulation of Neuroinflammation in Alzheimer's Disease

The physiological mechanisms (e.g., the growth and development of the neuron and glial cells) of the brain is epigenetically regulated [38, 39]. In general, epigenetics is a field of study that investigates the changes in the gene expression that do not involve the modification of a DNA sequence. In neurology, an epigenetic mechanism occurs in the central nervous system (CNS) as a response towards the presence of stimuli which normally act as mediators for the plasticity of neurons [40]. The epigenetic regulatory process takes place at both pre- and posttranscriptional levels in which during the pretranscriptional level it is mediated by DNA methylation and regulation of the chromatin structure. Conversely, at the posttranscriptional level, the noncoding RNAs (ncRNAs) act as the mediator [41].

It is estimated that over 600 different genes are involved in the pathogenesis of AD, in which environmental factors as well as the epigenomic aberrations contribute to such event [11, 42]. Amongst all the identified genes that are having a direct relationship with AD pathogenesis, apolipoprotein E (APOE) gene is said to be the predominant risk factor for late-onset AD (LOAD).

In 2008, Wang et al. investigated the difference between DNA methylation patterns in postmortem brains and lymphocytes from LOAD patients with the patterns found in healthy individuals by using base specific cleavage of single-stranded nucleic acids with MALDI-TOF mass spectroscopy analysis [43]. The study has demonstrated that the epigenetic distance increases by age which directly supports the role of epigenetics in the development of AD. The result from this study is parallel with the result obtained by Vijg et al. in which they observed that the patterns of methylation can become more random with age [44].

As a mammal ages, gradual hypomethylation and hypermethylation in its genome occur in most of the tissue and the promoter regions of genes, respectively [45, 46]. The hypomethylation results in genome instability due to the existence of repetitive sequences [46]. Tserel et al. have done a study to investigate age-related changes in DNA methylation and gene expression in CD4+ and CD8+ T cells between younger and older individuals [47]. Based on the result, they have detected the changes in DNA methylation in response to aging in T-lymphocytes in which most of the hypermethylated sites are located at CpG islands of silent genes and enriched for repressive histone marks. The changes could be due to the age-related process such as chronic antigen exposure that leads to proinflammatory phenotype [48].

Another example can be seen in a study conducted by Zawia et al. in which they managed to prove that the aberrant DNA methylation and disruption of the microRNA (miRNA) regulatory circuits are the key factors for the accumulation of A*β* [49]. The accumulation of A*β* promotes the production of reactive oxygen species (ROS) and eventually will cause the death of neurons due to the inhibition of DNA repair machinery mechanism by ROS [50].

Histone modification, on the other hand, has its centric roles in most biological processes which involve the manipulation and expression of DNA. It is a covalent posttranslational modification (PTM) to histone proteins that normally include acetylation, methylation, and phosphorylation. The mode of histone modification can be either via direct modification of the overall chromatin structure or via the modification of the effector molecule binding. The histone modifies the DNA package into a tight or loose chromatin structure allowing the accessibility of the transcriptional machinery to interact with the genes [51].

Histone acetylation is generally associated with the activation of DNA transcription. The process involves the regulation of chromatin dynamics and transcription, DNA replication and repair, gene silencing, and neuronal repression [52]. The level of histone acetylation is having an inverse relationship with age in which the level of histone acetylation will decrease as one ages [53]. In the context of AD, the level of histone acetylation will drastically decline as the disease progresses due to the inability of the transcription factors and DNA repair machinery to access the genes [54]. As a consequence, the number of synapses will decrease, followed by memory impairment and poor learning abilities [51].

## 4. Vitexin

Vitexin (apigenin-8-C-glucoside) is an active component found in abundance in most medicinal plant species such as pearl millet [55], hawthorn [56], bamboo [57], and *Ficus deltoidea* [58]. The compound is said to possess a number of pharmacological properties including anticancer [59], antinociceptive [60], antiviral [61], anti-inflammatory [62], and anti-AD [63]. However, this review will be focusing on the anti-AD and antineuroinflammatory properties that the vitexin compound possesses.

As explained in Figure 1, the accumulation of A*β* contributes to the progression and severity of AD. In a study conducted by Zhang et al., vitexin has shown its ability to limit the formation of A*β* via the inhibition of BACE1 enzyme [9]. BACE1 enzyme is a type of *β*-secretase enzyme that catalyses the proteolysis of APP molecules. The accumulation of A*β* will result in the dysfunction of proteasomes, generation of oxidized proteins, and subsequent aggregation of proteins. Such event will eventually stimulate the formation of reactive species and cause excitotoxicity [64].

Nurdiana et al. have conducted a study in the rat brain with diabetes induced by streptozotocin [9]. From the study, they found that 1 mg/kg of vitexin compound showed neuroprotective ability to the rat's brain by lowering the level of TBARS and lipid peroxidation products as well as improving the metabolism of glucose. In addition, the study also found out that the compound managed to improve the memory and learning skill of the rat upon being tested by using Morris' water maze technique.

Another example of vitexin's ability in conferring the neuroprotective effect can be found in a study done by Min et al. [30]. The study has used newborn C57BL/6 mice for the hypoxia-ischemia model. The mice have been pretreated with 30 and 60 mg/kg of vitexin compound. The study has discovered that the compound managed to significantly attenuate the volume of infarct (necrosis tissue), protect the cells against atrophy, and also improve the neurofunctional recovery of the mice [65].

Lyu et al. have investigated the effect of vitexin on HIF-1*α*, VEGF, and p38 MAPK protein expression in sevoflurane-induced newborn rat [66]. The result from this study revealed that treatment with vitexin managed to significantly suppress the expression of HIF-1*α*, VEGF, and p38 MAPK. From this finding, it shows that the compound is able to epigenetically modulate the expression of such proteins which are involved in the inflammatory pathway.

Recently, Krishnan and Kang concluded that vitexin managed to improve the behavior of zebrafish larvae upon treatment with vitexin [67]. In the study, the zebrafish larvae have been induced with 1 mM of acrylamide (ACR) 3 days post fertilization. 10 *μ*M of vitexin has been used to treat the ACR-induced zebrafish larvae. The results showed that vitexin is able to alleviate ACR-induced histological and behavioral changes in the zebrafish larvae. In addition, the authors also observed that vitexin managed to inhibit the CDK5 expression and hinder the expression of proinflammatory mediators. The expression of CDK5 is known to trigger the activation of microglial cells which sometimes leads to the hyperactivation of the cells.

## 5. Potential Use of Vitexin as Epigenetic Regulator in Neuroinflammation

In reference to Section 3 above, neuroinflammation can be epigenetically regulated by a number of stimuli present in its environment. Such stimuli are A*β* and proinflammatory cytokines that are found in abundance in the CNS environment. Vitexin has been the compound of interest amongst researchers recently since the discovery of its pharmacological properties of anti-inflammation [62] and anti-AD [63].

Accumulating number of evidences have shown that vitexin is capable of regulating the neuroinflammation activity which eventually might have the potential in regulating the progression of AD and other types of neurodegenerative diseases. As such, this section will be discussing the potential of vitexin in regulating the neuroinflammation activity focusing on the epigenetic mechanism aspect.

Malar et al. investigated the ability of vitexin to inhibit the toxicity of A*β*_25-35_ in Neuro-2a cells [68]. The cells have been pretreated with 50 *μ*M of vitexin prior to induction with A*β*_25-35_. The study found that the compound managed to inhibit the aggregation of A*β*_25-35_ and restore the viability of Neuro-2a cells by up to 92.86 ± 5.57%. In addition, the study also found that the compound managed to modulate the expression of genes that are involved in the antioxidant response mechanisms (*Nrf-2*, *HO-1*), cholesterol metabolism (*LXR-α*, *APOE*, *ABCA-1*, and *Seladin-1*), and endoplasmic reticulum stress (*Grp78*, *Gadd153*). The expression from these genes will lead to the neuroinflammation of the brain. Thus, the result indicates that the compound has the ability to attenuate the expression of certain genes without modifying the genes.

Next, Weyerer and Schaufele investigated the ability of vitexin to protect the brain against ischemia/reperfusion (I/R) injury [2]. The authors applied the focal cerebral I/R model in male Kunming mice which has been induced by middle cerebral artery occlusion (MCAO) for 2 hours followed by reperfusion for 22 hours. The result showed that vitexin is able to reduce the neurological deficit, cerebral infarct volume, and neuronal damage. From the Western Blot assay, it revealed that vitexin significantly upregulated the expression of p-ERK1/2 and downregulated p-JNK and p-p38. This indicates that vitexin is capable of regulating both mitogen-activated protein kinase (MAPK) and apoptosis signaling pathways. The MAPK signaling pathway is crucial as the pathway involves the regulation of proteins and cell functions related to proliferation, differentiation, survival, and death.

In support of Wang et al.'s (2015) findings, Rosa et al. also found that vitexin is not only capable of reducing the expression of proteins necessary for inflammatory pathways but also capable of reducing the migration of neutrophil when tested in Swiss-Webster mice that have been intraperitoneally challenged by ZY, CG, ƒMLP, and LPS inducers [69]. The reduction of neutrophil migration will reduce the inflammatory response at the inflammation site and thus reduce the chances of progression of neurodegeneration. In an *in vitro* study, the authors found that vitexin managed to attenuate the phosphorylation of p38, ERK1/2, and JNK proteins and hence postulated that the anti-inflammatory property of vitexin might be due to the inactivation of p38 kinases, ERK1/2, and JNK proteins [69].

## 6. Conclusion

The pathogenesis of AD is not limited to the neuronal compartment per sé. However, it is also involved with the immunological interaction in the brain [13]. The aggregated proteins (e.g., A*β* and *τ*) that interact with microglia will result in the activation of innate immune response which is characterized by the expression of proinflammatory cytokines (e.g., IL-1*β*, IL-6, and TNF-*α*). These proinflammatory cytokines will cause the neuroinflammation and eventually lead to the progression and severity of the disease.

There are a number of compounds that have been tested to tackle this situation. Recently, vitexin has been the compound of interest amongst researchers to investigate its ability in both preventing and treating AD. Nevertheless, the epigenetic mechanism by which vitexin regulates the expression of proinflammatory cytokines released by microglial cells is yet to be elucidated. Hence, this review has discussed the potential of vitexin as the candidate to epigenetically regulate the proinflammatory cytokines released by microglial cells.

From the previous studies, vitexin has shown promising preliminary results in regulating not only the expression of proinflammatory cytokines but also certain proteins (e.g., on HIF-1*α*, VEGF, and p38 MAPK proteins) that are involved in the inflammatory signaling pathways. Thus, we hope that the compound will be used as the new candidate to further study the epigenetic mechanism with regard to AD.
